# microRNA-153 Targets mTORC2 Component Rictor to Inhibit Glioma Cells

**DOI:** 10.1371/journal.pone.0156915

**Published:** 2016-06-13

**Authors:** Yan Cui, Jizong Zhao, Lei Yi, Yugang Jiang

**Affiliations:** 1 Department of Neurosurgery, the Second Xiangya Hospital of Central South University, Chang Sha, 410011,China; 2 Department of Neurosurgery, Tiantan Hospital, Capital Medical University, Beijing, 100050, China; Suzhou University, CHINA

## Abstract

Rictor upregulation and mTORC complex 2 (mTORC2) over-activation participate in glioma cell progression, yet the underling mechanisms are not known. We here identified microRNA-153 (miR-153) as a potential anti-Rictor miRNA, which was downregulated in multiple human glioma tissues and glioma cell lines (U87MG, T98G, U373MG and U251MG). miR-153 downregulation was correlated with Rictor (mRNA and protein) upregulation and p-Akt Ser473 (the mTORC2 indicator) over-activation in the glioma tissues and cells. Our *in vitro* evidences suggested that Rictor could be one primary target of miR-153 in glioma cells. Exogenous overexpression of miR-153 downregulated Rictor (mRNA and protein) and decreased p-Akt Ser473 in U87MG cells, leading to significant growth inhibition and apoptosis activation. Notably, U87MG cells with Rictor shRNA knockdown showed similar phenotypes of cells with miR-153 overexpression. More importantly, in Rictor-silenced U87MG cells, miR-153 expression failed to further affect cell growth nor apoptosis. *In vivo*, we showed that miR-153 overexpression dramatically inhibited U87MG tumor growth in nude mice. Together, these results suggest that miR-153 downregulation could be one important reason of Rictor upregulation and mTORC2 over-activation in glioma cells. Further, miR-153-induced anti-glioma cell activity is possibly via downregulating Rictor.

## 1. Introduction

Glioma causes large mortality around the world each year [1,2,3]. The prognosis for high-grade glioma (grade III-IV) has been poor [1,2,3]. In the past decades, postoperative radiation and temozolomide (TMZ) chemotherapy have become the standard treatment for glioma [4,5,6]. Yet, the overall survival has not been significantly improved for the affected patients [4,5,6]. One key hurdle is the molecular heterogeneity of glioma [1].

Mammalian target of rapamycin (mTOR) signaling is often dysregulated and hyper-activated in glioma, which mediates tumorigenesis, progression and chemoresistance [7,8,9]. mTOR lies in two distinct multi-protein mTOR complexes, including the traditional mTOR complex 1 (mTORC1) and later-discovered rapamycin-insensitive mTOR complex 2 (mTORC2) [10,11]. mTORC2 is composed of mTOR, Rictor (rapamycin-insensitive companion of mTOR), mSIN1 (mammalian stress-activated protein kinase-interacting protein 1), mLST8 and other components [7,8,9]. Rictor is a key component of mTORC2 and is required for mTORC2 activation [12,13]. It directly associates with two other mTORC2 components, mSIN1 and Protor1, to form the mTORC2 complex [12,13]. Rictor depletion would block mTORC2 activation [12,13]. Existing evidences have demonstrated that Rictor overexpression and mTORC2 over-activation promote glioma cell migration and proliferation [14,15]. The underlying mechanism of Rictor overexpression has not been studied. In the current study, we focused on the role on microRNA (miRNA) in the process.

miRNAs are capable of regulating gene expression at both translational and post-transcriptional levels [16,17]. These 19–24 nucleotide single-stranded noncoding RNAs silence targeted mRNAs through partial complementarity in their 3′ untranslated regions (UTRs) [16,17]. Studies have demonstrated that miRNAs are dysregulated in gliomas, which positively regulate a number of cancerous behaviors [18]. In the current study, we identified a potential anti-Rictor miRNA: microRNA-153 (miR-153). Our results suggest that miR-153 downregulation could be the reason of Rictor upregulation and mTORC2 over-activation in human glioma cells. Overexpression of miR-153-induced anti-glioma cell activity is possibly via downregulating Rictor.

## 2. Material and Methods

### 2.1. Reagents and antibodies

Rictor (sc-271081) and (β-) tubulin (sc-55529) antibodies were purchased from Santa Cruz (Santa Cruz, CA). p-Akt Ser473 (#9271) and Akt1 (#2967) antibodies were obtained from Cell Signaling Tech (Danvers, MA). Puromycin was purchased from Sigma (Shanghai, China). All the cell culture reagents were obtained from Gibco (Shanghai, China).

### 2.2. Culture of glioma cell lines

Established human glioma cells (U87MG, T98G, U373MG and U251MG lines) were maintained in DMEM/RPMI medium, supplemented with 10% fetal bovine serum (FBS) and antibiotics, and in the CO_2_ incubator at 37°C.

### 2.3. Culture of human primary astrocytes

Human primary astrocyte cultures were purchased from the iBS cell bank of Fudan University (Shanghai, China). The astrocytes were derived from the cerebral cortices of a single trauma patient. Ninety eight percent or more of the astrocytes were positive of glial fibrillary acidic protein (GFAP). Primary human astrocytes were maintained in astrocyte media (Science Cell, Carlsbad, CA) containing 10% FBS, 1% astrocyte growth supplement and 1% Penicillin/Streptomycin. All investigations involving clinical samples were conducted according to the principles expressed in the Declaration of Helsinki and national regulations. The Scientific Ethical Committee of Central South University approved the use of human tissues for primary cell culture or gene detection (ID: 2014-03-112). Written informed consent was obtained from each participant.

### 2.4. Human glioma tissues

Fresh human glioma tissue specimens were obtained from nine informed-consent glioma patients at the time of surgery. The patients’ basic parameters were: Male: 6/Female: 3; 42–59 years old; Grade II: 5/Grade III: 4. Fresh tumor specimens were minced, homogenized and dissolved via the tissue lysis buffer (Biyuntian, Wuxi, China). Samples were then subjected to real-time PCR assay or Western blotting assay. All investigations involving clinical samples were conducted with the guidance described above.

### 2.5. MTT assay of cell growth

Glioma cells (3 × 10^3^ per well) were seeded onto 96-well plates for 48 hours. After treatment of cells, MTT tetrazolium salt (0.5 mg/ml, Sigma) was added for 3 hours. Thereafter, 150 μl of DMSO per well was added to dissolve formazan crystals. The absorbance was tested by a plate reader at a test wavelength of 490 nm.

### 2.6. Clonogenicity assay of cell growth

Glioma cells (0.5 × 10^5^ per dish) were suspended in complete medium with 0.1% agar (Sigma), which was then added on the top of a pre-solidified 100 mm culture dish. The cell culture medium was renewed every two days for a total of ten days. Afterwards, the number of visible colonies was manually counted.

### 2.7. Histone-DNA Enzyme-linked immunosorbent assay (ELISA) assay

Glioma cell apoptosis was examined by Histone-DNA ELISA PLUS kit (Roche Applied Science, Shanghai, China) according to the manufacturer’s protocol [19].

### 2.8. Western blotting

Cells or fresh tissues were lysed with the lysis buffer as described [19]. Cell extracts were clarified by centrifugation at 10,000 *g* for 15 min. Twenty-five μg proteins per sample were analyzed on a 10% SDS-page gel [19]. Afterwards, samples were transferred onto polyvinylidene fluoride (PVDF) membranes (Millipore, Shanghai, China), which were then blocked with blocking solution [19], followed by incubation with the primary antibody and corresponding second antibody. The detection was performed by Super-signal West Pico Enhanced Chemiluminescent (ECL) Substrate. The intensity of each band was quantified via ImageJ software, and the value was normalized to corresponding equal loading [20].

### 2.9. RNA extraction and real-time PCR

RNA was extracted with TRIZOL reagents according to standard procedures, and was reverse-transcribed. The PCR reaction mixture contained 1× SYBR Master Mix (Applied Biosystem, Foster City, CA), 1 μg RNA and 200 nM primers. An ABI Prism 7300 Fast Real-Time PCR system (Foster City, CA) was applied for PCR reactions. mRNA expression was quantified using the ^ΔΔ^Ct method. GAPDH served as the internal control. The GAPDH primers were described in Chen’s study [21]. The Rictor primers were also described previously [22]. For miRNA analysis, real-time PCR was performed using PrimeScript miRNA RT-PCR Kit (Takara) according to the manufacturer’s instructions. The miR-153 primers were described early [23]. All the primers and sequences were synthesized by OriGene (Beijing, China).

### 2.10. miR-153 overexpression

Pre-miR-153 (see sequence in [23]) was sub-cloned into pSuper-puromycin vector (a gift from Dr. Tian [24]) to generate miR-153 expression construct. For transfection, glioma cells were seeded onto 6-well plates at 50–60% confluence, which were then transfected with miR-153 construct (0.25 μg/ml) via Lipofectamine 2000 reagents (Invitrogen, Shanghai, China). After 36 hours of incubation, cells were cultured in puromycin-containing complete medium for a total of 8 days. miR-153 expression in the stable cells was tested by real-time PCR assay. Control cells were transfected with non-sense microRNA-control (“miR-C”) (a gift from Dr. Tian [24]).

### 2.11. Rictor shRNA knockdown and stable cell selection

The Rictor shRNA lentiviral particles were purchased from Santa Cruz Biotech (sc-61478-V, Santa Cruz, CA). The lentiviral shRNA (10 μl/ml medium) was added to the cultured U87MG cells. After 36 hours, cell cultured medium was replaced by the puromycin-containing complete medium. The medium was renewed every 2–3 days until single resistant colony was formed (3–4 weeks). Rictor expression in the stable colony was detected by Western blotting. Control cells were infected with same concentration of lentiviral scramble control shRNA (sc-108080, Santa Cruz), and were also subjected to same puromycin selection.

### 2.12. Xenograft assay

Stable U87MG cells bearing miR-153 or miR-C were subcutaneously (s.c.) injected into the right flanks of 4-week-old female nude mice (each mouse: 2 × 10^6^ cells in 200 µl of Matrigel). We initiated the recording when the tumor volume reached around 100 mm^3^. The tumor volumes and mice body weights were recorded weekly. Volumes were calculated via the formula: π/6×width ^2^× length. Estimated average daily tumor growth was also calculated. Mice survival was recorded at week-7. The animal protocol was approved by the Central South University’s Institutional Animal Care and Use Committee (IACUC, ID: 2014-03-25) and Ethics committee. Animals were observed on daily bases. Humane endpoints were defined as a loss of more that 15% of body mass, a tumor greater than 1.5 cm, or inability to ambulate or rise for food and water. If animals reached these endpoints they were euthanized by exsanguination. Animal surgery and euthanasia using decapitation were performed under Hypnorm/Midazolam anesthesia, and all efforts were made to minimize suffering.

### 2.13. Statistical analysis

The data presented in this study were means ± standard deviation (SD). Statistical differences were analyzed by one-way *ANOVA* followed by multiple comparisons performed with post hoc Bonferroni test (SPSS). Values of *p* < 0.05 were considered statistically significant.

## 3. Results

### 3.1. miR-153 downregulation correlates with Rictor upregulation in multiple human glioma tissues and cell lines

**Fig 1:**
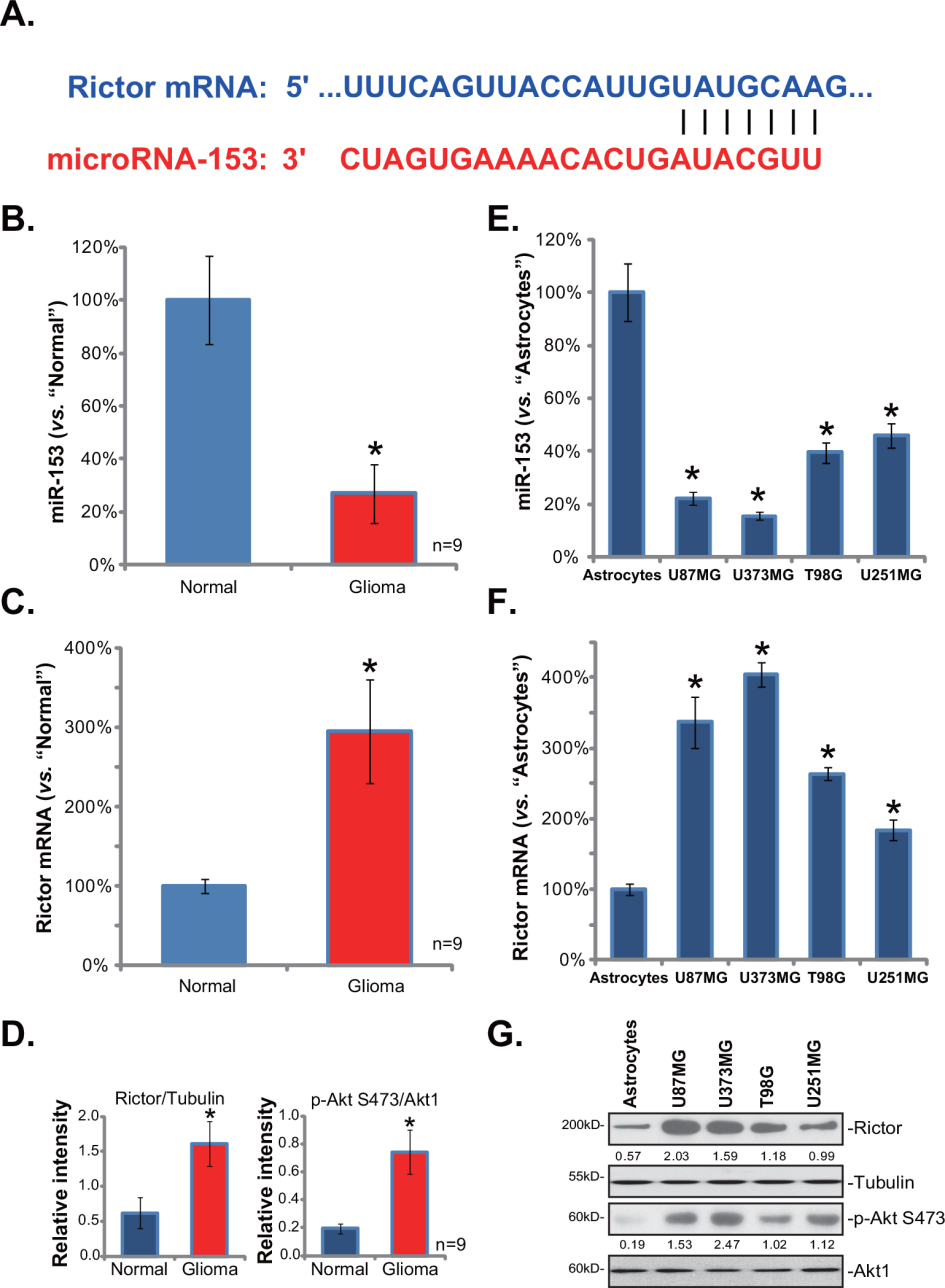
miR-153 downregulation correlates with Rictor upregulation in multiple human glioma tissues and cell lines. miR-153 (-3p) and its putative binding sequence in the 3’-UTR of Rictor mRNA (A). Expressions of miR-153 (B), Rictor mRNA (C) as well as Rictor protein (D, vs. Tubulin) and p-Akt (D, vs. Akt1) in human glioma tissues (“Glioma”) and their surrounding normal brain tissues (“Normal”) were shown. Expressions of miR-153 (E), Rictor mRNA (F) as well as Rictor protein (G) and p-Akt (G) in primary human astrocytes (“Astrocytes”) and established glioma cell lines (T98G,U373MG, U251MG and U87MG) were shown. Rictor protein expression (vs. Tubulin) and p-Akt Ser473 (vs. Akt1) were quantified (D and G). Experiments in this figure were repeated three times, with similar results obtained. Bars stand for mean ± SD. * *p* < 0.05 vs. “Normal” group (B-D, n = 9). * *p* < 0.05 vs. “Astrocytes” group (E-F, n = 3).

First, we show that microRNA-153 (miR-153) selectively targets the 3’ untranslated regions (UTRs) of Rictor mRNA (Fig 1A). Next, its expression in human glioma tissues was examined. A total of nine pairs of fresh human glioma tissues and their surrounding normal tissues were collected. Real-time PCR results demonstrated that miR-153 level was dramatically downregulated in glioma tissues (“Glioma”), as compared to its level in the surrounding normal brain tissues (“Normal”) (Fig 1B). On the other hand, Rictor mRNA level was increased in the human glioma tissues (Fig 1C). Correspondingly, Rictor protein expression and mTORC2 activity (indicated by p-Akt Ser473) were upregulated (Fig 1D).

Next, we tested miR-153 and Rictor expressions in human glioma cells. A total of four established human glioma cell lines were included: U87MG, T98G, U373MG and U251MG. As compared to the primary human astrocytes (“Astrocytes”), miR-153 level was significantly reduced in all four lines of glioma cells (Fig 1E), but the Rictor mRNA level was increased (Fig 1F). Rictor protein expression was also higher in these glioma cell lines, along with p-Akt Ser473 (Fig 1G). Note that miR-153 downregulation and Rictor upregulation were most dramatic in U87MG cells and U373MG cells (Fig 1E–1G), these two cell lines were chosen for further studies.

### 3.2. Rictor is a target of miR-153 in glioma cells

**Fig 2:**
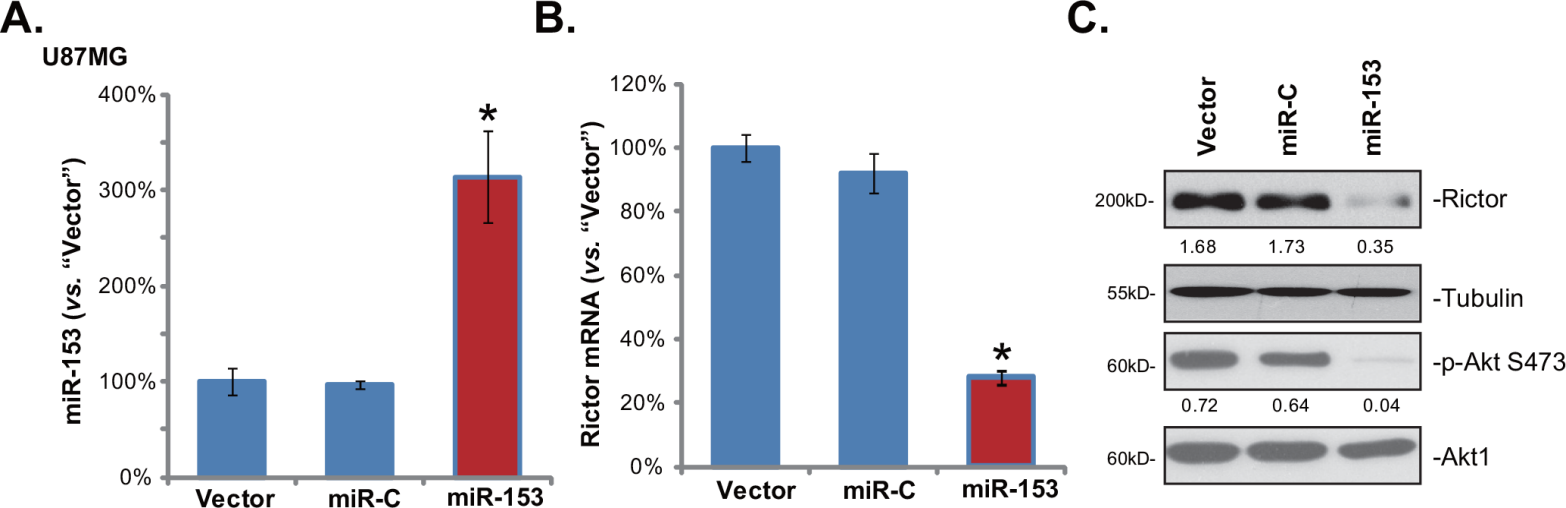
Rictor is a target of miR-153 in glioma cells. Stable U87MG cells expressing miR-153, microRNA-control (“miR-C”) or empty vector (“pSuper-puro”) were subjected to real-time PCR assay of miR-153 (A) and Rictor mRNA (B). Expressions of listed proteins in these cells were also tested (C). Rictor protein expression (vs. Tubulin) and p-Akt Ser473 (vs. Akt1) were quantified (C). Bars stand for mean ± SD. * *p* < 0.05 vs. “Vector” group (A and B, n = 5).

We next wanted to know if miR-153 downregulation was the reason of Rictor upregulation in glioma cells. pre-miR-153 construct was introduced into U87MG cells to establish miR-153-expressing cell line (See Methods). Real-time PCR results in Fig 2A confirmed miR-153 overexpression in the stable U87MG cells. miR-153 expression clearly downregulated Rictor mRNA in U87MG cells (Fig 2B). As a result, Rictor protein expression and p-Akt Ser473 level were also decreased (Fig 2C). Expectably, microRNA-control (“miR-C”) showed no effect on Rictor nor p-Akt Ser473 in U87MG cells (Fig 2A–2C). Similar results were also observed in U373MG cells (Data not shown). These results indicated that miR-153 downregulation could be the reason of Rictor upregulation and mTORC2 over-activation in glioma cells.

### 3.3. miR-153 overexpression inhibits glioma cell growth, and activates cell apoptosis

**Fig 3:**
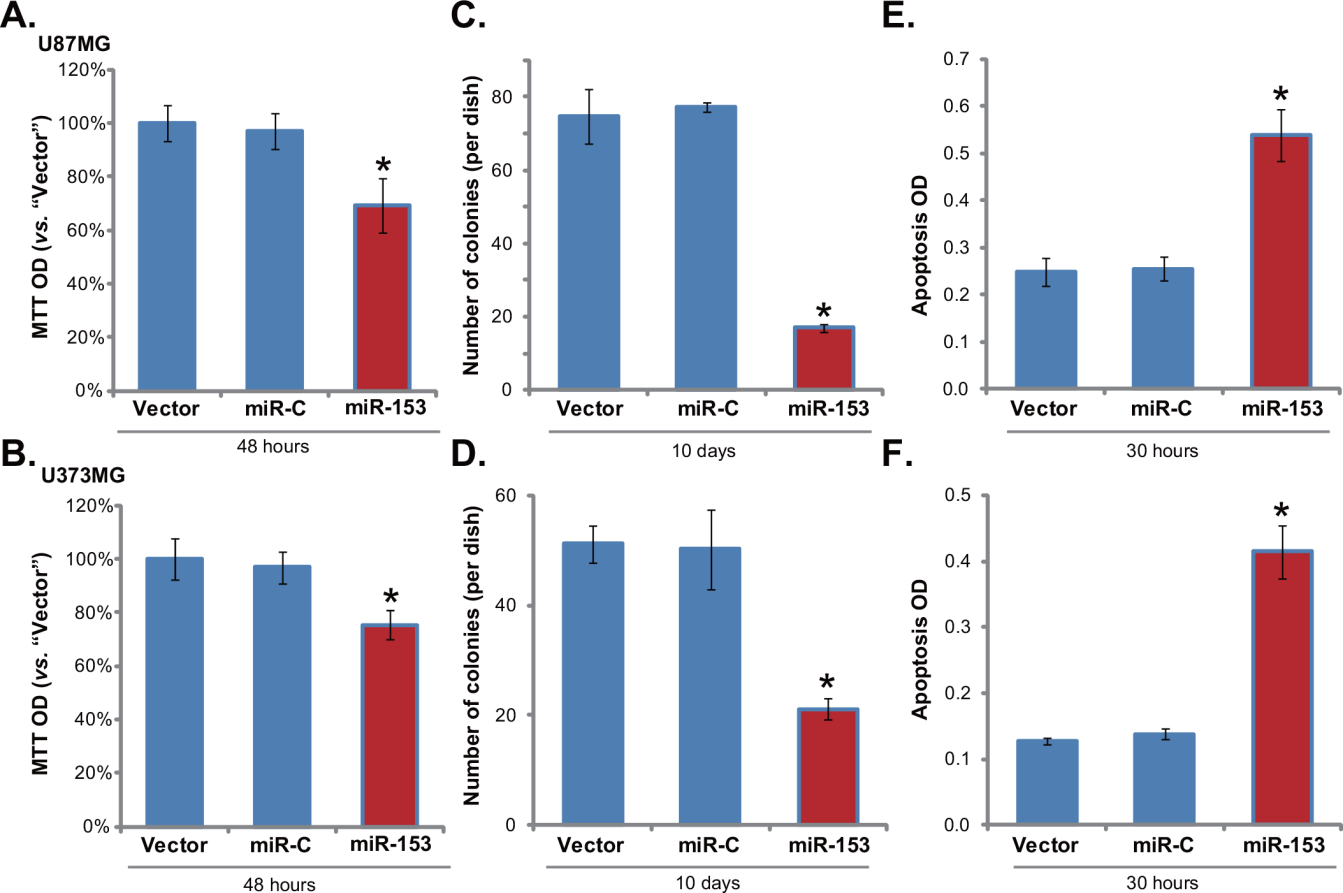
miR-153 overexpression inhibits glioma cell growth, and activates cell apoptosis. Exact same number of stable glioma cells (U87MG or U373MG lines) expressing miR-153, microRNA-control (“miR-C”) or empty vector (“pSuper-puro”) were subjected to MTT assay (A and B) or clonogenicity assay (C and D) to analyze of cell growth; Apoptosis level in these cells was also tested by Histone DNA ELISA assay (E and F). Bars stand for mean ± SD. * *p* < 0.05 vs. “miR-C” group (n = 6).

The results above suggest that Rictor is a target gene of miR-153, and overexpression of miR-153 will lead to Rictor downregulation and p-Akt Ser473 inhibition in glioma cells. Since Rictor upregulation and mTORC2 over-activation are important for glioma cell growth and apoptosis-resistance [15], we then tested the potential role of miR-153 on glioma cell functions. First, MTT results in Fig 3A and 3B showed that miR-153 overexpression significantly inhibited growth of U87MG and U373MG cells. Meanwhile, the number of U87MG/U373MG colonies was decreased sharply after forced miR-153 expression (Fig 3C and 3D). When analyzing cell apoptosis, we detected a clear apoptosis activation in miR-153-expressing U87MG cells (Fig 3E) and U373MG cells (Fig 3F). These results indicated that miR-153 overexpression exerted anti-growth and pro-apoptosis activity against glioma cells.

### 3.4. miR-153-induced anti-glioma cell activity is mediated via downregulating Rictor

**Fig 4:**
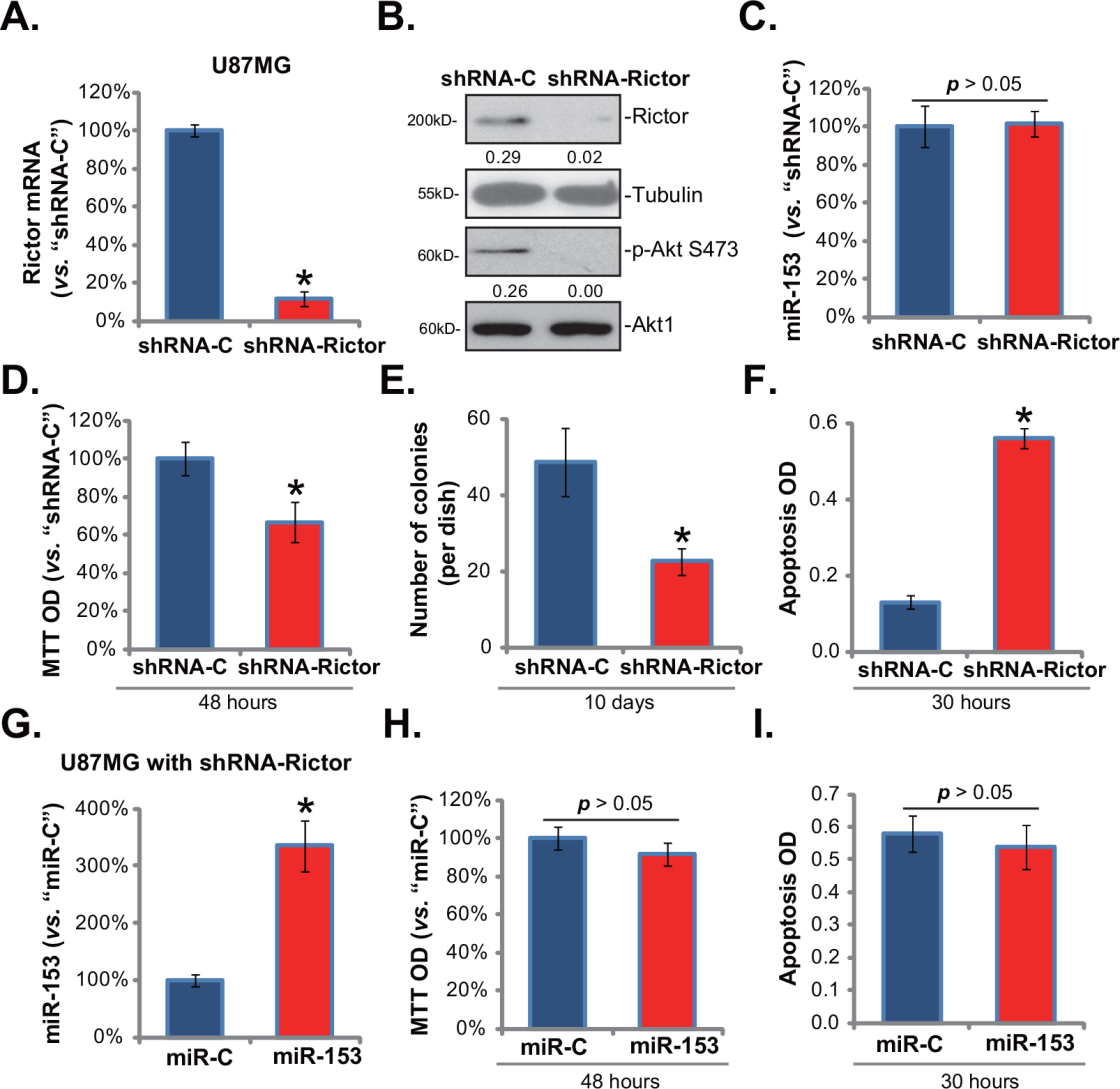
miR-153-induced anti-glioma cell activity is mediated via downregulating Rictor. Relative Rictor mRNA (A) and miRNA-153 (C) expression in stable U87MG cells with scramble control shRNA (“shRNA-C”) or Rictor shRNA (“shRNA-Rictor”) was tested by Real-time PCR assay. Expressions of listed proteins in these cells were also shown (B). Same number of U87MG cells with “shRNA-C” or “shRNA-Rictor” were subjected to MTT assay (D) and clonogenicity assay (E) to test cell growth; Cell apoptosis was also tested (Histone DNA ELISA assay, F). Rictor shRNA-expressing stable U87MG cells were transfected with miR-153 or microRNA-control (“miR-C”), miR-153 expression (G, Real-time PCR assay), cell growth (E, MTT assay) and apoptosis (F, Histone DNA ELISA assay) in these cells were tested. Rictor expression (vs. Tubulin) and p-Akt Ser473 (vs. Akt1) were quantified (B). Bars stand for mean ± SD. * *p* < 0.05 vs. “shRNA-C” group (A, C, D-F, n = 5). * *p* < 0.05 vs. “miR-C” group (G, n = 5).

Thus far, we have shown that miR-153 downregulated Rictor and inhibited glioma cell growth *in vitro*. Next, we analyzed the link between the two. First, the shRNA strategy was applied to knockdown Rictor in U87MG cells, and the stable cell line was established by puromycin selection (See Methods). Real-time PCR assay confirmed Rictor mRNA depletion by the targeted shRNA (Fig 4A). Consequently, Rictor protein expression and p-Akt Ser473 were largely decreased in stable U87MG cells (Fig 4B). The level of miR-153 was obviously not affected by Rictor shRNA (Fig 4C). Note that the MTT OD (Fig 4D) and the number of viable colonies (Fig 4E) were both decreased in Rictor shRNA-expressing U87MG cells, indicating cell growth inhibition. Further, Rictor shRNA induced apoptosis activation in U87MG cells, which was detected by the Histone DNA ELISA assay (Fig 4F). These results showed that cells with Rictor shRNA showed similar phenotypes (growth inhibition and apoptosis activation) as cells with miR-153 overexpression. If Rictor is the main target of miR-153 in glioma cells, miR-153’s activity against glioma cells should be diminished in Rictor-silenced cells. To test this hypothesis, we again overexpressed miR-153 in Rictor-silenced U87MG cells (Fig 4G). Indeed, forced expression of miR-153 failed to further affect U87MG cell growth (Fig 4G) or apoptosis (Fig 4I) in these cells. These results indicate that miR-153 over-expression-mediated anti-glioma cell activity is likely mediated via downregulating Rictor.

### 3.5. The anti-glioma activity by miR-153 *in vivo*

**Fig 5:**
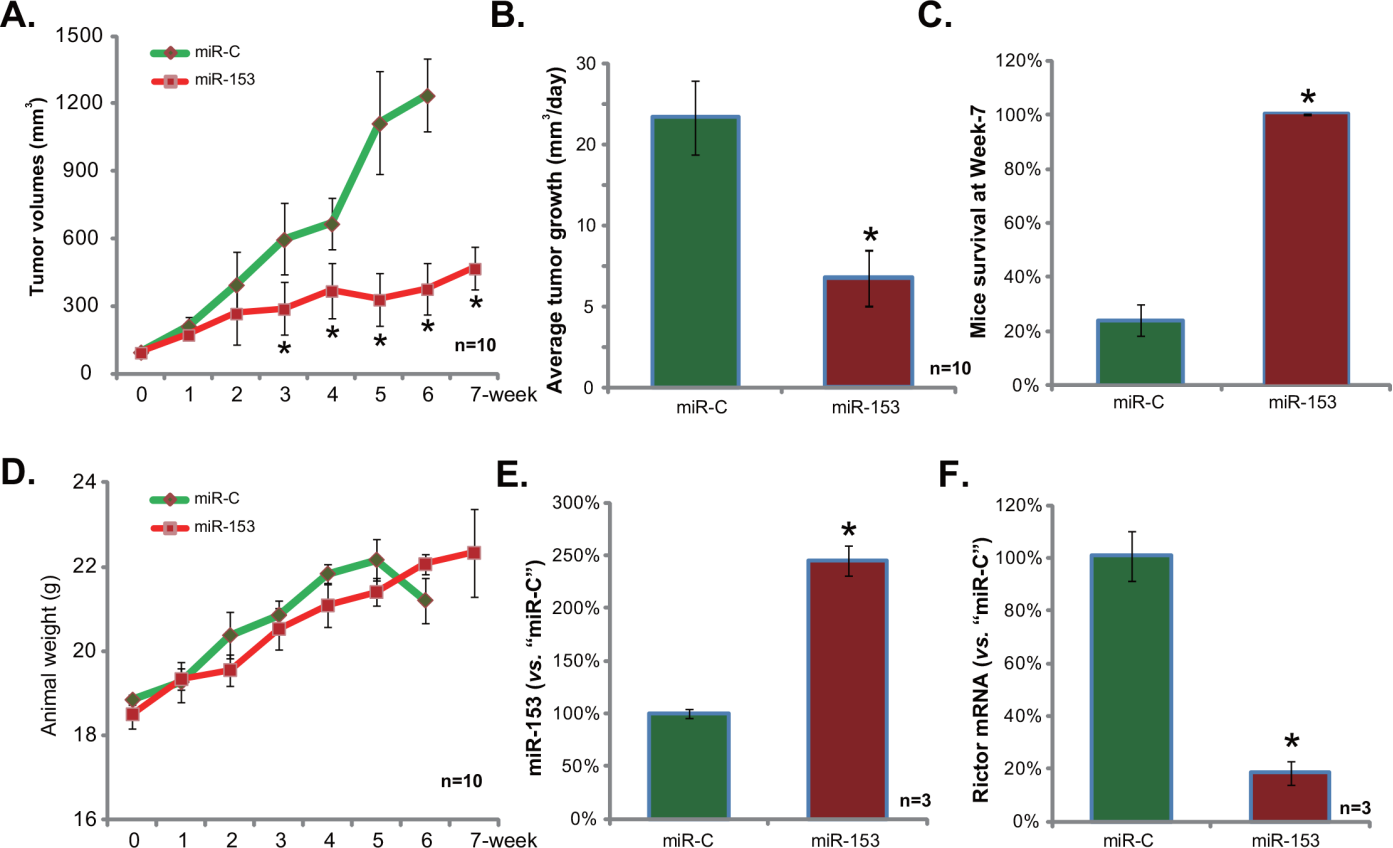
The anti-glioma activity by miR-153 *in vivo*. miR-153-expressing U87MG cells (“miR-153”) or miR-C-expressing U87MG cells (“miR-C”) were inoculated into the nude mice (13 mice per group), tumor volumes (A) and mice body weights (D) were recorded weekly; Estimated daily tumor growth was calculated (B); Mice survival at week-7 was also presented (C, summarizing of three-set repeats). At week-5, three xenografted U87MG tumors per group were isolated, miR-153 (E) and Rictor mRNA (F) expressions in the fresh tissues were tested by real-time PCR assay; Bars stand for mean ± SD. * *p* < 0.05 vs. “miR-C” tumor group (A-F).

Finally, we tested miR-153’s activity on glioma cell growth *in vivo*. Stable U87MG cells (2 × 10^6^ cells per mouse) expressing miR-153 or miR-C were s.c. injected into the nude mice, and xenografted tumors were established. Fig 5A showed that the average volume of miR-153-expressing U87MG tumors was much smaller than that of the miR-C-expressing U87MG tumors. Average daily tumor growth results further confirmed the anti-glioma activity by miR-153 *in vivo* (Fig 5B). At week-7, the mice bearing miR-153 U87MG tumors were all alive, but the majority of mice with miR-C tumors were already dead (Fig 5C). Notably, the average mice body weight was not significantly different between the two groups (Fig 5D). We also analyzed expression of miR-153 and Rictor in the U87MG tumors. Real-time PCR results confirmed miR-153 upregulation (Fig 5E) and Rictor mRNA depletion (Fig 5F) in miR-153-expressing tumors (at Week-5, n = 3 for each). Together, these results demonstrated that miR-153 over-expression inhibited U87MG tumor growth *in vivo*.

## 4. Discussions

The high-grade glioma is among the most aggressive malignancy with an average survival of less than 12–14 months [2,3,25]. The rapamycin-insensitive mTORC2 is composed of mTOR, mLST8, mSin1, and Rictor, which has shown functions in cell survival, proliferation and actin cytoskeleton [26,27,28,29]. mTORC2 servers as the upstream kinase for Akt at Ser473, and is required for Akt fully activation [26,27,28,29]. It has been shown that a large proportion of human gliomas have Rictor upregulation, mTORC2 hyperactivity, and high level of p-Akt [14,15]. In line with these findings, we showed that Rictor expression (mRNA and protein) and p-Akt Ser473 were both upregulated in the tested human glioma tissues and cell lines. Significantly, shRNA-mediated knockdown of Rictor potently decreased p-Akt Ser473 and inhibited glioma cell growth. These results indicated that Rictor could be an important oncogene for glioma.

miR-153 was first discovered as one of the several brain-specific miRNAs, based on analysis of expression profile of over one hundred miRNAs in adult organs [30]. Recent evidences have indicated that miR-153 was dramatically downregulated in several cancer cells [31,32]. Further, in grade IV (GBM multiforme) human gliomas, miR-153 expression appeared to be depleted [31,32]. These results [31,32] have implied that miR-153 could be a tumor suppressor.

One key finding of this study is that Rictor might be a key target gene of miR-153 in glioma cells. In human glioma tissues and cells, miR-153 downregulation was negatively correlated with Rictor upregulation and mTORC2 (p-Akt Ser473) over-activation. Importantly, exogenous overexpression of miR-153 downregulated Rictor and decreased p-Akt Ser473 in glioma cells. Meanwhile, significant growth inhibition and apoptosis activation were observed in the miR-153-expressing glioma cells. Importantly, our *in vivo* studies showed that miR-153 downregulated Rictor/p-Akt Ser473, and potently inhibited U87MG tumor growth in nude mice. Intriguingly, in Rictor-silenced glioma cells, miR-153 expression failed to further decrease cell growth or increase cell apoptosis. Therefore, Rictor should be the primary target of miR-153 in mediating its anti-glioma cell activity.

## 5. Conclusions

Together, we showed that miR-153 downregulation could be the reason of Rictor upregulation and mTORC2 over-activation in glioma cells. Further, miR-153-induced anti-glioma cell activity is possibly through downregulating Rictor.

## References

[1] Westphal M, Lamszus K (2011) The neurobiology of gliomas: from cell biology to the development of therapeutic approaches. Nat Rev Neurosci 12: 495–508. 10.1038/nrn3060 21811295

[2] Siegel R, Naishadham D, Jemal A (2012) Cancer statistics, 2012. CA Cancer J Clin 62: 10–29. 10.3322/caac.20138 22237781

[3] Siegel R, Ma J, Zou Z, Jemal A (2014) Cancer statistics, 2014. CA Cancer J Clin 64: 9–29. 10.3322/caac.21208 24399786

[4] Khasraw M, Lassman AB (2009) Neuro-oncology: late neurocognitive decline after radiotherapy for low-grade glioma. Nat Rev Neurol 5: 646–647. 10.1038/nrneurol.2009.194 19953115

[5] Pollack IF (2010) Neuro-oncology: Therapeutic benefits of reirradiation for recurrent brain tumors. Nat Rev Neurol 6: 533–535. 10.1038/nrneurol.2010.144 20927054

[6] Wang Y, Jiang T (2013) Understanding high grade glioma: molecular mechanism, therapy and comprehensive management. Cancer Lett 331: 139–146. 10.1016/j.canlet.2012.12.024 23340179

[7] Huang TT, Sarkaria SM, Cloughesy TF, Mischel PS (2009) Targeted therapy for malignant glioma patients: lessons learned and the road ahead. Neurotherapeutics 6: 500–512. 10.1016/j.nurt.2009.04.008 19560740 PMC3600166

[8] Lefranc F, Rynkowski M, DeWitte O, Kiss R (2009) Present and potential future adjuvant issues in high-grade astrocytic glioma treatment. Adv Tech Stand Neurosurg 34: 3–35. 19368079 10.1007/978-3-211-78741-0_1

[9] Li X, Wu C, Chen N, Gu H, Yen A, Cao L, et al (2016) PI3K/Akt/mTOR signaling pathway and targeted therapy for glioblastoma. Oncotarget. 10.18632/oncotarget.7961 PMC5078108 26967052

[10] Zaytseva YY, Valentino JD, Gulhati P, Evers BM (2012) mTOR inhibitors in cancer therapy. Cancer Lett 319: 1–7. 10.1016/j.canlet.2012.01.005 22261336

[11] Laplante M, Sabatini DM (2012) mTOR signaling in growth control and disease. Cell 149: 274–293. 10.1016/j.cell.2012.03.017 22500797 PMC3331679

[12] Sarbassov DD, Ali SM, Kim DH, Guertin DA, Latek RR, Erdjument-Bromage H, et al (2004) Rictor, a novel binding partner of mTOR, defines a rapamycin-insensitive and raptor-independent pathway that regulates the cytoskeleton. Curr Biol 14: 1296–1302. 15268862 10.1016/j.cub.2004.06.054

[13] Jacinto E, Facchinetti V, Liu D, Soto N, Wei S, Jung SY, et al (2006) SIN1/MIP1 maintains rictor-mTOR complex integrity and regulates Akt phosphorylation and substrate specificity. Cell 127: 125–137. 16962653 10.1016/j.cell.2006.08.033

[14] Bashir T, Cloninger C, Artinian N, Anderson L, Bernath A, Holmes B, et al (2012) Conditional astroglial Rictor overexpression induces malignant glioma in mice. PLoS One 7: e47741 10.1371/journal.pone.0047741 23077666 PMC3471885

[15] Masri J, Bernath A, Martin J, Jo OD, Vartanian R, Funk A, et al (2007) mTORC2 activity is elevated in gliomas and promotes growth and cell motility via overexpression of rictor. Cancer Res 67: 11712–11720. 18089801 10.1158/0008-5472.CAN-07-2223

[16] Du H, Guo L, Fang F, Chen D, Sosunov AA, McKhann GM, et al (2008) Cyclophilin D deficiency attenuates mitochondrial and neuronal perturbation and ameliorates learning and memory in Alzheimer's disease. Nat Med 14: 1097–1105. 10.1038/nm.1868 18806802 PMC2789841

[17] Huang AL, Ostrowski MC, Berard D, Hager GL (1981) Glucocorticoid regulation of the Ha-MuSV p21 gene conferred by sequences from mouse mammary tumor virus. Cell 27: 245–255. 6277498 10.1016/0092-8674(81)90408-6

[18] Moller HG, Rasmussen AP, Andersen HH, Johnsen KB, Henriksen M, Duroux M (2013) A systematic review of microRNA in glioblastoma multiforme: micro-modulators in the mesenchymal mode of migration and invasion. Mol Neurobiol 47: 131–144. 10.1007/s12035-012-8349-7 23054677 PMC3538124

[19] Qin LS, Jia PF, Zhang ZQ, Zhang SM (2015) ROS-p53-cyclophilin-D signaling mediates salinomycin-induced glioma cell necrosis. J Exp Clin Cancer Res 34: 57 10.1186/s13046-015-0174-1 26024660 PMC4486428

[20] Qin LS, Yu ZQ, Zhang SM, Sun G, Zhu J, Xu J, et al (2013) The short chain cell-permeable ceramide (C6) restores cell apoptosis and perifosine sensitivity in cultured glioblastoma cells. Mol Biol Rep 40: 5645–5655. 10.1007/s11033-013-2666-4 24065522

[21] Chen MB, Zhou ZT, Yang L, Wei MX, Tang M, Ruan TY, et al (2016) KU-0060648 inhibits hepatocellular carcinoma cells through DNA-PKcs-dependent and DNA-PKcs-independent mechanisms. Oncotarget. 10.18632/oncotarget.7742 PMC4941370 26933997

[22] Li J, Ren J, Liu X, Jiang L, He W, Yuan W, et al (2015) Rictor/mTORC2 signaling mediates TGFbeta1-induced fibroblast activation and kidney fibrosis. Kidney Int 88: 515–527. 10.1038/ki.2015.119 25970154 PMC4558569

[23] Liang C, Zhu H, Xu Y, Huang L, Ma C, Deng W, et al (2012) MicroRNA-153 negatively regulates the expression of amyloid precursor protein and amyloid precursor-like protein 2. Brain Res 1455: 103–113. 10.1016/j.brainres.2011.10.051 22510281

[24] Chen MB, Yang L, Lu PH, Fu XL, Zhang Y, Zhu YQ, et al (2015) MicroRNA-101 down-regulates sphingosine kinase 1 in colorectal cancer cells. Biochem Biophys Res Commun 463: 954–960. 10.1016/j.bbrc.2015.06.041 26071354

[25] Chen W, Zheng R, Baade PD, Zhang S, Zeng H, Bray F, et al (2016) Cancer statistics in China, 2015. CA Cancer J Clin. 10.3322/caac.21338 26808342

[26] Sabatini DM (2006) mTOR and cancer: insights into a complex relationship. Nat Rev Cancer 6: 729–734. 16915295 10.1038/nrc1974

[27] Sparks CA, Guertin DA (2010) Targeting mTOR: prospects for mTOR complex 2 inhibitors in cancer therapy. Oncogene 29: 3733–3744. 10.1038/onc.2010.139 20418915 PMC3031870

[28] Sun SY (2013) mTOR kinase inhibitors as potential cancer therapeutic drugs. Cancer Lett 340: 1–8. 10.1016/j.canlet.2013.06.017 23792225 PMC3779533

[29] Duzgun Z, Eroglu Z, Biray Avci C (2016) Role of mTOR in glioblastoma. Gene 575: 187–190. 10.1016/j.gene.2015.08.060 26341051

[30] Sempere LF, Freemantle S, Pitha-Rowe I, Moss E, Dmitrovsky E, Ambros V (2004) Expression profiling of mammalian microRNAs uncovers a subset of brain-expressed microRNAs with possible roles in murine and human neuronal differentiation. Genome Biol 5: R13 15003116 10.1186/gb-2004-5-3-r13 PMC395763

[31] Gaur A, Jewell DA, Liang Y, Ridzon D, Moore JH, Chen C, et al (2007) Characterization of microRNA expression levels and their biological correlates in human cancer cell lines. Cancer Res 67: 2456–2468. 17363563 10.1158/0008-5472.CAN-06-2698

[32] Xu J, Liao X, Wong C (2010) Downregulations of B-cell lymphoma 2 and myeloid cell leukemia sequence 1 by microRNA 153 induce apoptosis in a glioblastoma cell line DBTRG-05MG. Int J Cancer 126: 1029–1035. 10.1002/ijc.24823 19676043

